# ST-segment elevation myocardial infarction in older adults: echocardiographic characterization and outcomes in contemporary practice: STEMI in older patients

**DOI:** 10.1007/s10554-026-03775-w

**Published:** 2026-07-11

**Authors:** Laima Caunite, Rinchyenkhand Myagmardorj, Xavier Galloo, Dorien Laenens, Jan Stassen, Takeru Nabeta, Idit Yedidya, Maria Chiara Meucci, Jurrien H. Kuneman, Inge J. van den Hoogen, Sophie E. van Rosendael, Hoi W. Wu, Victor M. van den Brand, Adrian Giuca, Karlis Trusinskis, Nina Ajmone Marsan, Jeroen J. Bax, Pieter van der Bijl

**Affiliations:** 1 Department of Cardiology, Leiden University Medical Center, Albinusdreef 2, Leiden, 2333 ZA The Netherlands; 2 Department of Cardiology, Vrije Universiteit Brussel (VUB), Universitair Ziekenhuis Brussel (UZ Brussel), Brussels, Belgium; 3 Department of Cardiology, Sint Maarten Hospital, Mechelen, Belgium; 4 Department of Cardiology, Jessa Hospital, Hasselt, Belgium; 5 Department of Cardiology, Rabin Medical Center, Petah-Tikva, Israel; 6 Faculty of Medicine, Tel-Aviv University, Tel-Aviv, Israel; 7 Department of Cardiovascular Science, Fondazione Policlinico Universitario A. Gemelli IRCCS, Rome, Italy; 8 Latvian Cardiology Center, Pauls Stradins Clinical University Hospital, Riga, Latvia; 9 Faculty of Medicine and Life Sciences, University of Latvia, Riga, Latvia; 10 Heart Centre, University of Turku, Turku University Hospital, Turku, Finland

**Keywords:** older patients, myocardial infarction, echocardiography, cardiac function, left ventricular remodeling, guideline directed medical therapy

## Abstract

Evidence on cardiac remodeling, left ventricular (LV) function recovery, medication use and outcomes in older ST-segment elevation myocardial infarction (STEMI) patients is limited. We examined these aspects at baseline and evolution one year post-infarct in patients aged ≥ 80 versus < 80.STEMI registry data were retrospectively analyzed. The older subgroup included patients aged ≥ 80 years at index hospitalization. Echocardiography was performed at baseline and one year after STEMI. LV remodeling was defined as > 20% relative increase in the LV end-diastolic volume. One-year cardiovascular outcomes and five-year all-cause mortality are reported.Study included 2178 patients; 132 (6.1%) were aged ≥ 80. Older patients had worse baseline LV ejection fraction (47.2 ± 9.6% versus 49.8 ± 8.2%; *p* < 0.001) and experienced more adverse LV remodeling (29% versus 19%; *p* = 0.024). Nevertheless, LV function improved similarly in both groups. Beta-blocker and renin-angiotensin-aldosterone system inhibitor use was lower (86.4% versus 94.2%; *p* < 0.001 and 90.2% versus 96.5%; *p* < 0.001, respectively) but diuretic prescription higher (18.2% versus 8.1%; *p* < 0.001) in older patients. One year post-infarct beta-blocker and renin-angiotensin-aldosterone system inhibitor use decreased and was similar in both groups, but older patients continued to receive more diuretics and suffered higher heart failure hospitalization rates (7.0% versus 2.6%; *p* = 0.006). Five-year mortality was 25.8% in older versus 4.3% in younger patients (log-rank χ2 133.2; *p* < 0.001).Older patients experienced more adverse LV remodeling, received less beta-blockers and renin-angiotensin-aldosterone system inhibitors but more diuretics at discharge and one year, compared to younger patients. Nevertheless, LV function recovered similarly in both groups.

**Supplementary Information:**

The online version contains supplementary material available at https://doi.org/10.1007/s10554-026-03775-w.

## Introduction

With globally increasing longevity, the population ≥ 80 years old is projected to triple from 143 million in 2019 to 426 million in 2050 [1]. Since the incidence of cardiovascular disease increases with age [2], the proportion of older patients having a myocardial infarction (MI) is expected to grow in parallel [3]. Older patients having a MI are characterized by a distinct risk profile [3, 4] and experience high rates of bleeding, heart failure, reinfarction and death [4–6].

Data have consistently shown that older patients have worse left ventricular (LV) systolic and diastolic function after MI [4, 7–9]. However, most previous studies included a high proportion of non-ST-segment elevation MI (NSTEMI) [9] and non-invasive treatment strategies were frequently employed [8, 9]. Detailed information on cardiac function and post-infarct remodeling remains scarce in this age group [8, 10].

Older patients have either been excluded, underrepresented or demonstrated inconclusive results [11–15] in the historical landmark trials that shaped the contemporary treatment of STEMI. Observational data indicate a wide variation in renin-angiotensin system inhibitor, beta-blocker and statin prescriptions on discharge post-MI in this population [7, 8, 16, 17]. Furthermore, few studies have explored how prescription practices change over time [8].

We therefore aimed to evaluate the echocardiographic findings at baseline and one year post-infarct in patients aged ≥ 80 years versus < 80 years, and compare medication prescription practices between these age groups. In addition, we analyzed the prevalence of heart failure hospitalization, MI, new-onset atrial fibrillation, stroke, ventricular tachyarrhythmias, out-of-hospital cardiac arrest and pacemaker implantation.

## Methods

### Patient population

Patients presenting with STEMI, who were referred for primary percutaneous coronary intervention between 2006 and 2021 at the Leiden University Medical Center (The Netherlands) were included from an ongoing registry (MISSION! ) [18]. All patients were treated according to contemporary European Society of Cardiology guidelines [3]. Clinical, angiographic and survival data from baseline and the pre-planned one year outpatient visit were collected from the hospital information system (EPD-Vision; Leiden University Medical Center, Leiden, The Netherlands), which is linked to municipal registries. Per protocol, comprehensive transthoracic echocardiography was performed within 48 h of the index hospitalization and at the one year outpatient visit post-STEMI. The older patient subgroup included those aged ≥ 80 years at the time of index hospitalization. The study investigated cardiovascular events (heart failure hospitalization, MI, new-onset atrial fibrillation, stroke, ventricular tachyarrhythmias, out-of-hospital cardiac arrest and pacemaker implantation) within the first year, as well as five year all-cause mortality.

All data used in the present study were collected for routine clinical purposes and handled anonymously. For retrospective analysis of clinically acquired data, the institutional review board waived the need for patient written informed consent.

### Transthoracic echocardiography

All patients underwent transthoracic echocardiography in the left lateral decubitus position with a commercially available echocardiography system (Vivid 7, Vivid E9, or E95; GE Vingmed Ultrasound, Horten, Norway). Images were acquired and archived for off-line analysis (EchoPac 202, 203 and 204, GE Vingmed Ultrasound, Horten, Norway). LV end-diastolic diameter, LV end-systolic diameter, interventricular septal thickness and LV posterior wall thickness were measured from the parasternal long-axis view. LV mass was calculated by the cube formula from the parasternal long axis view [19] and indexed for body surface area. LV end-diastolic end-systolic volumes were measured and LV ejection fraction (LVEF) was calculated using the biplane Simpson’s method from apical four- and two-chamber views [19]. LV global longitudinal strain (LVGLS) was averaged from apical four-chamber, two-chamber and three-chamber views and measured with two-dimensional speckle tracking. LVGLS values are presented as absolute values. LV remodeling was defined as a relative increase in the LV end-diastolic volume of > 20% one year post-MI [20]. Left atrial volume was measured from apical four- and two-chamber views according to Simpson’s biplane method and indexed for body surface area. LV peak early (E) diastolic velocity was measured using pulsed-wave Doppler traces of transmitral flow. E′ was measured using tissue Doppler imaging at the septal and lateral mitral annulus from the apical four-chamber view and E/E’ calculated. Tricuspid annular plane systolic excursion was measured from the apical four-chamber view. Right ventricular fractional area change was derived from right ventricular end-diastolic and end-systolic areas, obtained from the focused right ventricular apical view. Pulmonary artery systolic pressure was estimated using the peak velocity of the tricuspid regurgitation jet according to the simplified Bernoulli equation, adding the right atrial pressure, estimated by the diameter and inspiratory collapse of the inferior vena cava. Valvular dysfunction was evaluated according to the current recommendations [21] and the severity reported as a categorical variable.

### Statistical analysis

Continuous data are presented as mean ± standard deviation or median and interquartile range, as appropriate. Categorical data are presented as frequencies and percentages. Continuous variables were compared with independent samples t-tests if normally distributed or Mann-Whitney U tests if non-normally distributed. Categorical variables were compared with chi-square tests or the Fisher exact test. Changes in echocardiographic parameters over time within the groups were compared using the paired t-test for continuous variables and the McNemar test for categorical variables. Changes over time between the groups were compared using linear mixed models for continuous variables and general linear mixed models for categorical variables. Survival differences between the groups were demonstrated by Kaplan-Meier curves and compared using the log-rank test. Statistical analysis was performed using SPSS version 29.0 (IBM, Armonk, New York, USA). All statistical tests were two-sided, and a *p* value < 0.05 was considered statistically significant.

## Results

The study population included 2178 patients (mean age 61 ± 12 years; 75% male). Baseline characteristics are shown in Table 1. A total of 132 patients (6.1%) were aged ≥ 80 years. This group included a higher proportion of women, had more comorbidities, worse kidney function, lower diastolic blood pressure, a higher heart rate and a trend towards higher troponin levels on admission when compared to younger patients, who had a slightly higher body mass index and four-times higher prevalence of smoking.

**Table 1:** Baseline clinical characteristics of the study population, stratified according to the age groups

Parameter	Overall population (*N* = 2178)	≥ 80-year-olds (*N* = 132)	< 80-year-olds (*N* = 2046)	*p* value
Age, years	60.9 ± 11.7	83.1 ± 2.9	59.5 ± 10.5	< 0.001
Male	1638 (75.2%)	78 (59.1%)	1560 (76.2%)	< 0.001
Arterial hypertension	839 (38.6%)	77 (59.2%)	762 (37.3%)	< 0.001
Hyperlipidemia	504 (23.2%)	31 (23.7%)	473 (23.2%)	0.90
Previous MI	131 (6%)	12 (9.1%)	119 (5.8%)	0.13
History of CHF	25 (1.1%)	4 (3.0%)	21 (1%)	0.036
Previous AF	47 (2.2%)	9 (6.8%)	38 (1.9%)	< 0.001
Family history of CAD	937 (43.5%)	20 (15.4%)	917 (45.3%)	< 0.001
Diabetes mellitus	234 (10.8%)	22 (16.7%)	212 (10.4%)	0.024
Prior statin use	382 (17.6%)	28 (21.7%)	354 (17.4%)	0.21
Current smoker	862 (39.6%)	14 (10.6%)	848 (41.4%)	< 0.001
COPD	84 (3.9%)	11 (8.3%)	73 (3.6%)	0.006
BMI, kg/m^2^	26.8 ± 4.1	26.1 ± 4.1	26.9 ± 4	0.022
Peak troponin T, ng/l	3080 (1230; 6320)	3747 (1790; 7935)	2990 (1198; 6240)	0.09
eGFR (CKD-EPI 2021), ml/min/1.73m^2^	93 (78; 102)	69 (54; 80)	94 (80; 103)	< 0.001
LM/LAD as culprit	974 (44.7%)	64 (48.5%)	910 (44.5%)	0.37
Number of vessels diseased				< 0.001
One	939 (43.1%)	31 (23.5%)	908 (44.4%)	
Two	724 (33.2%)	46 (34.8%)	678 (33.1%)	
Three	515 (23.6%)	55 (41.7%)	460 (22.5%)	
Multivessel disease	1239 (56.9%)	101 (76.5%)	1138 (55.6%)	< 0.001
Systolic blood pressure on admission, mmHg	135 ± 25	137 ± 28	135 ± 25	0.25
Diastolic blood pressure on admission, mmHg	81 ± 16	77 ± 16	82 ± 16	< 0.001
Heart rate at discharge, bpm	69 ± 12	72 ± 13	69 ± 12	0.002
QRS > 120 ms	135 (6.2%)	25 (18.9%)	110 (5.4%)	< 0.001
LBBB present	24 (1.1%)	6 (4.5%)	18 (0.9%)	< 0.001

Data are presented as mean ± standard deviation, median (interquartile range) and n (%). Abbreviations: AF: atrial fibrillation; BMI: body mass index; bpm: beats per minute; CAD: coronary artery disease; CHF: chronic heart failure; CKD-EPI: Chronic Kidney Disease Epidemiology Collaboration; COPD: chronic obstructive pulmonary disease; eGFR: estimated glomerular filtration rate; LAD: left anterior descending artery; LBBB: left bundle branch block; LM: left main stem; MI: myocardial infarction

### Baseline echocardiographic findings

The baseline echocardiographic results are summarized in Table 2. Older patients had smaller LV end-diastolic dimension, greater LV mass and worse LV systolic function, with lower LVEF and LVGLS. Furthermore, higher LV filling pressures, greater left atrial volumes, increased pulmonary artery systolic pressures and worse right ventricular systolic function (measured by tricuspid annular plane systolic excursion) were noted in older patients. Severe valvular pathology was infrequent in the study population. Altogether, 19 (0.9%) patients had severe mitral regurgitation, 12 (0.6%) severe tricuspid regurgitation and two patients in each group had severe aortic stenosis.

**Table 2:** Baseline and one year-follow-up echocardiographic characteristics, stratified according to the age groups

Parameter	Overall population (*N* = 2178) baseline	Overall population (*N* = 1980) follow-up	≥ 80-year-olds (*N* = 132) baseline	≥ 80-year-olds (*N* = 86) follow-up	*p* value	< 80-year-olds (*N* = 2046) follow-up	< 80-year-olds (*N* = 1894) follow-up	*p* value	*p* value for baseline measurements	*p* value for change between groups
LVEDD, mm	49 ± 5.5	50 ± 5.7	47 ± 6	49 ± 6.2	0.15	49 ± 5.4	50 ± 5.7	< 0.001	< 0.001	0.77
LVESD, mm	32 ± 6.4	32 ± 7.2	31 ± 6.9	32 ± 7.4	0.25	32 ± 6.4	32 ± 7.2	0.1	0.25	0.3
IVST, mm	11.3 ± 1.6	10.8 ± 1.5	11.9 ± 1.5	11.6 ± 1.6	0.4	11.2 ± 1.6	10.8 ± 1.5	< 0.001	< 0.001	0.17
PWT, mm	9.9 ± 1.4	9.5 ± 1.3	10 ± 1.5	9.5 ± 1.3	0.027	9.9 ± 1.4	9.5 ± 1.3	< 0.001	0.41	0.99
LVEDV, ml	136 ± 45	141 ± 48	122 ± 47	132 ± 44	0.001	137 ± 45	142 ± 48	< 0.001	< 0.001	0.064
LV remodeling, %		388 (19.6%)		25 (29.1%)			363 (19.2%)			0.024*
LVEDVI, ml/ m^2^	68 ± 21	71 ± 22	65 ± 23	71 ± 22	< 0.001	69 ± 21	71 ± 22	< 0.001	0.10	0.039
LVESV, ml	69 ± 29	68 ± 31	65 ± 34	66 ± 31	0.35	70 ± 29	68 ± 31	< 0.001	0.11	0.079
LVESVI, ml/ m^2^	35 ± 14	34 ± 15	35 ± 17	35 ± 16	0.33	35 ± 14	34 ± 15	< 0.001	0.96	0.052
LVMI, ml/ m^2^	97 ± 21	94 ± 21	101 ± 22	102 ± 26	0.9	96 ± 21	94 ± 21	< 0.001	0.013	0.15
LVEF, %	49.7 ± 8.3	53.2 ± 7.9	47.2 ± 9.6	51.9 ± 9.2	< 0.001	49.8 ± 8.2	53 ± 7.9	< 0.001	< 0.001	0.77
LVEF < 40%	279 (12.8%)	121 (6.1%)	28 (21.5%)	8 (9.3%)	0.033	251 (12.3%)	113 (6.0%)	< 0.001	0.002	0.85
LVGLS, %	13.9 ± 3.6	16 ± 3.4	12.9 ± 4	15.1 ± 3.8	< 0.001	14.0 ± 3.6	16 ± 3.3	< 0.001	0.001	0.3
LVGLS < 16.0%	1477 (69.0%)	869 (44.6%)	96 (76.8)	47 (55.3%)	< 0.001	1381 (68.5%)	822 (44.1)	< 0.001	0.053	0.41
E/E’	10.9 ± 4.2	9.5 ± 4.2	13.8 ± 5.3	11.8 ± 5.0	0.004	10.7 ± 4.1	9.4 ± 4.2	< 0.001	< 0.001	0.02
E/A	0.99 ± 0.42	0.97 ± 0.42	0.81 ± 0.48	0.75 ± 0.4	0.87	1 ± 0.41	0.98 ± 0.41	0.07	< 0.001	0.62
LAVI, ml/ m^2^	28.3 ± 9.8	30.6 ± 10.3	32.5 ± 14	36.5 ± 14.2	< 0.001	28.1 ± 9.5	30.4 ± 10	< 0.001	< 0.001	< 0.001
PASP, mm Hg	26 ± 10	24.5 ± 9.3	30 ± 11	31.6 ± 13.2	0.54	26 ± 10	24.2 ± 8.9	0.39	< 0.001	0.1
RVEDA, cm^2^	22.1 ± 6.2	23.6 ± 6.5	20.8 ± 5.8	21.8 ± 6.2	0.042	22.2 ± 6.2	23.7 ± 6.5	< 0.001	0.013	0.48
RVESA, cm^2^	12.7 ± 4.3	13.2 ± 4.3	12.3 ± 4.4	12.7 ± 4.5	0.062	12.7 ± 4.3	13.2 ± 4.3	< 0.001	0.25	0.71
RVFAC, %	43 ± 8.4	44 ± 7.9	42 ± 8.2	43 ± 7	0.9	43 ± 8.4	44 ± 8	< 0.001	0.51	0.36
TAPSE, mm	20 ± 3.1	20.6 ± 3.2	19.2 ± 3.4	19.6 ± 2.5	0.53	20 ± 3.1	21 ± 3.2	0.002	0.006	0.31
Severe MR	19 (0.9%)	21 (1.1%)	2 (1.5%)	1 (1.2%)	> 0.99	17 (0.9%)	20 (1.1%)	0.18	0.42	0.50
Severe TR	12 (0.6%)	10 (0.5%)	3 (2.3%)	1 (1.2%)	0.56	9 (0.4%)	9 (0.5%)	0.59	0.006	0.38

Data are presented as mean ± standard deviation and n (%). * p value for comparison between the groups on follow-up.Abbreviations: IVST: interventricular septal thickness; LAVI: left atrial volume index; LVEDD: left ventricular end-diastolic diameter; LVEDV: left ventricular end-diastolic volume; LVEDVI: left ventricular end-diastolic volume index; LVEF: left ventricular ejection fraction; LVESD: left ventricular end-systolic diameter; LVESV: left ventricular end-systolic volume; LVESVI: left ventricular end-systolic volume index; LVGLS: left ventricular global longitudinal strain; LVMI: left ventricular mass index; MR: mitral regurgitation; PASP: pulmonary artery systolic pressure; PWT: posterior wall thickness; RVEDA: right ventricular end-diastolic area; RVESA: right ventricular end-systolic area; RVFAC: right ventricular fractional area change; TAPSE: tricuspid annular plane systolic excursion; TR: tricuspid regurgitation

### Discharge medication and follow-up

**Fig. 1:**
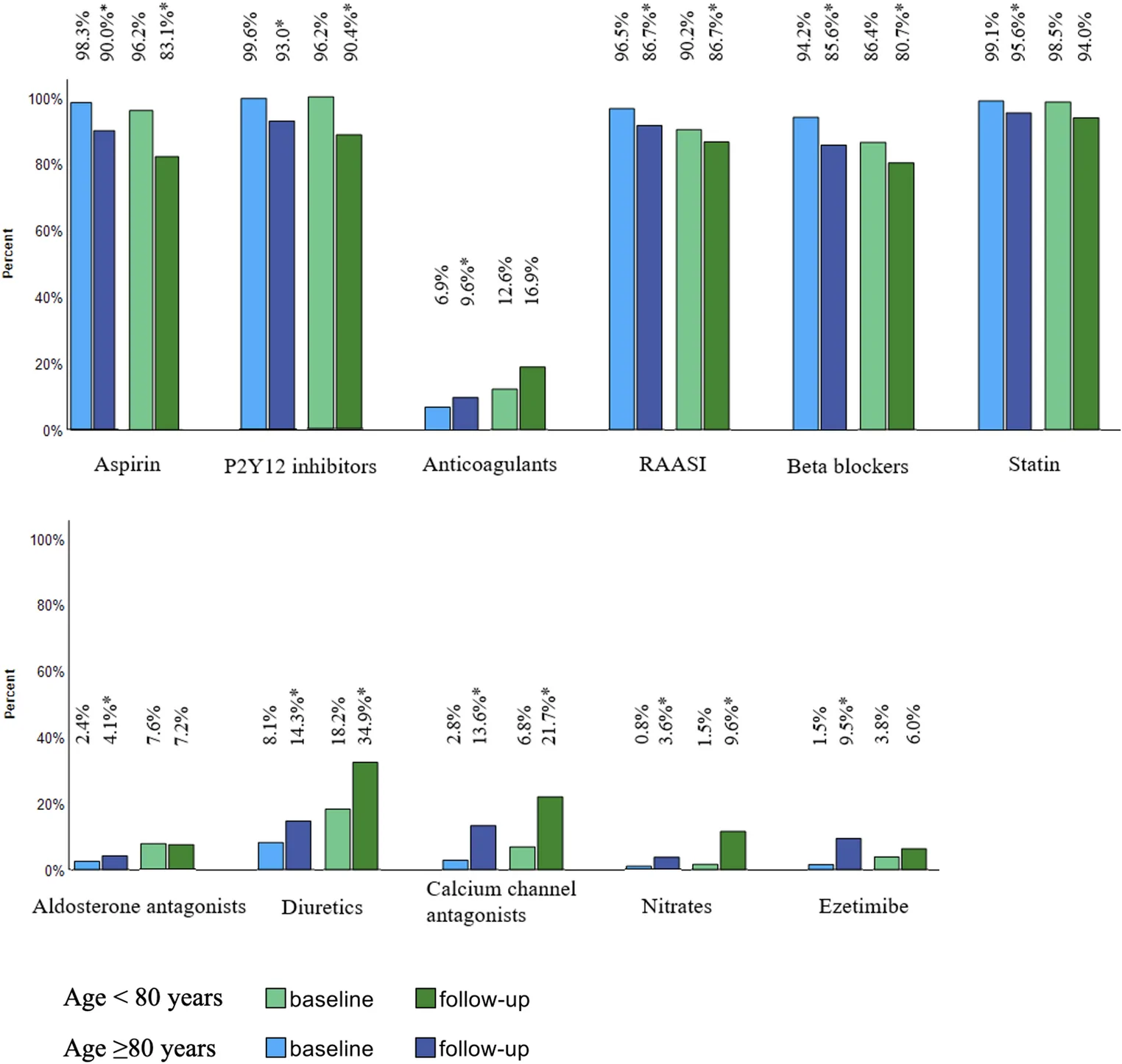
Medication at hospital discharge and at one year stratified according to the age groups Abbreviations: RAASI: renin-angiotensin-aldosterone system inhibitors. *p < 0.05 for change within the group

Discharge medication is summarized in Fig. 1. Anticoagulation was prescribed more frequently in older patients, while the use of triple antithrombotic therapy was similar between the two groups. Older patients received less anti-remodeling pharmacotherapy, namely renin-angiotensin-aldosterone system inhibitors and beta-blockers, but were prescribed more aldosterone antagonists, calcium channel antagonists and diuretics.

198 (9.1%) patients did not undergo a follow-up echocardiogram, which was more commonly encountered in older versus younger patients (35% versus 7.4%, χ2 112.8; *p* < 0.001).

Patients aged ≥ 80 years who were lost to follow-up were older, had higher rates of atrial fibrillation and a trend towards lower LVEF when compared to the subgroup who did undergo follow-up echocardiography (Supplemental Table 1).

### Evolution of cardiac function and dimensions at one year post-STEMI

One year follow-up echocardiography was available in 1980 (91%) patients (Table 2). Older patients experienced higher rates of adverse LV remodeling, compared to younger individuals. Nonetheless, both groups demonstrated a similar improvement in LVEF and LVGLS at follow-up. At one year, the percentage of patients with LVEF below 40% was similar between the two groups (*p* = 0.21), although LVGLS below 16% was more common in the older patients (*p* = 0.04).

LV filling pressure improved to a greater extent in older patients, but the increase in left atrial volume index was also more pronounced in this group.

### Medication prescription changes at one year

At one year follow-up, a decrease in the prescription of antiplatelet agents, renin-angiotensin-aldosterone system inhibitors, beta-blockers and statins was observed in both groups (Fig. 1). The younger patient group received more often aspirin, while older patients received more often anticoagulants, diuretics and nitrates.

### Outcomes

**Fig. 2:**
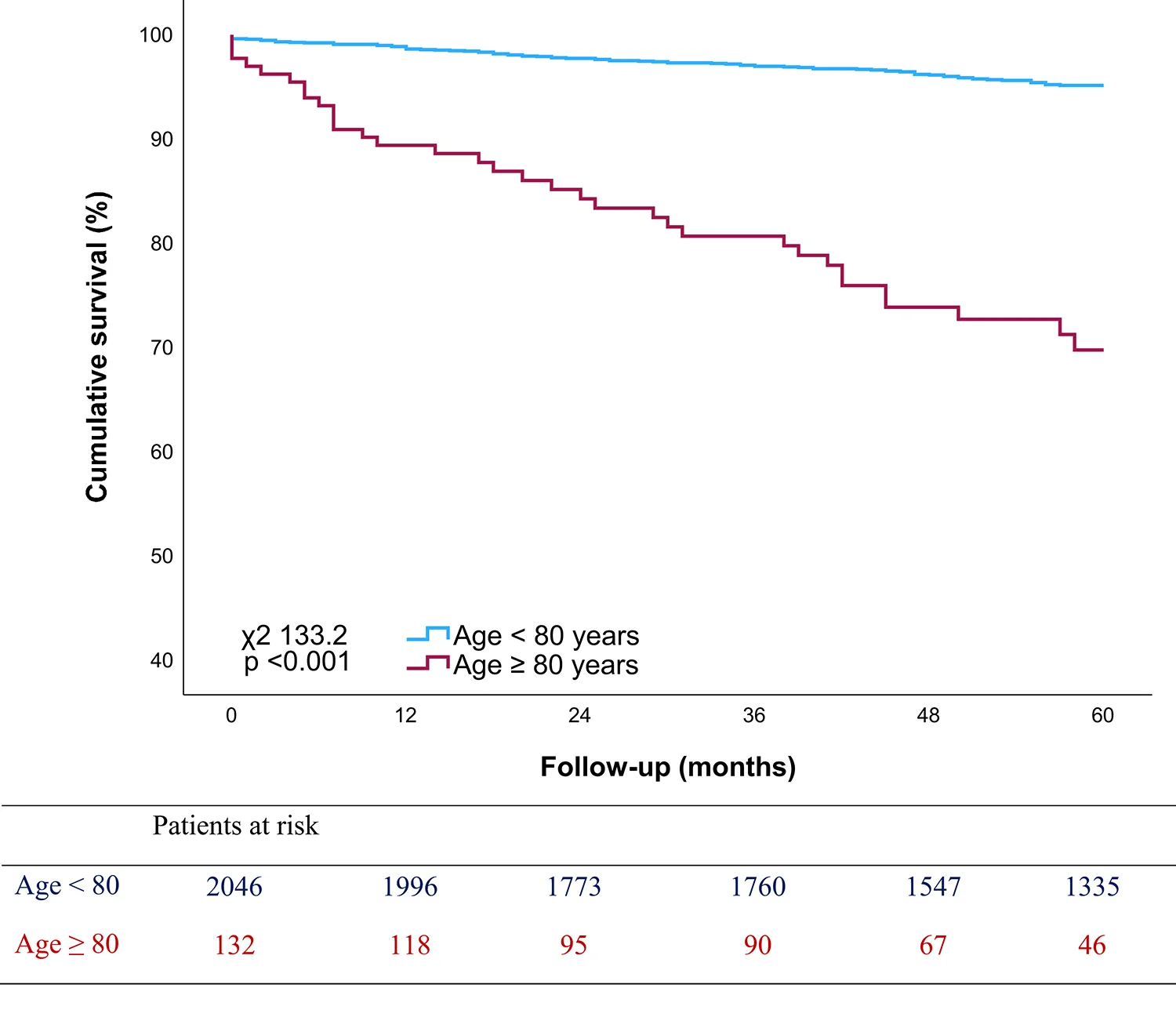
Kaplan-Meier curve for five-year all-cause mortality, stratified according to the age groups

During the first year after STEMI, older patients experienced higher rates of heart failure hospitalization, new-onset atrial fibrillation, pacemaker implantation and a trend towards more strokes, in comparison to younger patients (Table 3). During a median follow-up of 60 (47; 60) months, 122 patients died. One- and five-year mortality was 14 (10.6%) and 34 (25.8%) in older patients versus 24 (1.2%) and 88 (4.3%) in younger patients (log-rank χ2 133.2; *p* < 0.001; Fig. 2).

**Table 3:** One year outcomes stratified according to the age groups

Character	Overall population (*N* = 2178)	≥ 80-year-olds (*N* = 132)	< 80-year-olds (*N* = 2046)	*p* value
All-cause mortality	38 (1.7%)	14 (10.6%)	24 (1.2%)	< 0.001
Heart failure hospitalization	60 (2.9%)	8 (7.0%)	52 (2.6%)	0.006
Recurrent myocardial infarction	43 (2.1%)	1 (0.9%)	42 (2.1%)	0.36
New-onset atrial fibrillation	136 (6.5%)	17 (14.9%)	119 (6.0%)	< 0.001
Stroke				0.059
Ischemic stroke	18 (0.9%)	2 (1.8%)	16 (0.8%)	
Hemorrhagic stroke	3 (0.1%)	1 (0.9%)	2 (0.1%)	
Ventricular tachyarrhythmias	26 (1.2%)	2 (1.8%)	24 (1.2%)	0.61
Out-of-hospital cardiac arrest	5 (0.2%)	0	5 (0.2%)	0.76
Pacemaker implantation	10 (0.5%)	3 (2.6%)	7 (0.4%)	< 0.001

Data are presented as n (%)

## Discussion

The present study explored echocardiographic characteristics, cardiac remodeling and medication prescription practices in older patients. The main findings can be summarized as follows: (1) while patients aged ≥ 80 had worse left and right ventricular function at the index hospitalization, suffered more LV remodeling and a greater increase in left atrial volume, they demonstrated a similar improvement in LV systolic function, compared to younger patients (< 80 years), (2) older patients were prescribed less beta-blockers and renin-angiotensin-aldosterone system inhibitors at discharge but received more diuretics, (3) while at one year post-MI the prescription rates of anti-remodeling pharmacotherapy (beta-blockers and renin-angiotensin aldosterone inhibitors) were similar in both groups, older patients continued to receive more symptom-targeted medication and suffered higher rates of heart failure hospitalizations and (4) although the five-year all-cause mortality was six times higher in the older patient group, the overall survival rates remained relatively good, considering the high risk profile of these patients.

### Cardiac function in older MI patients

Older patients more frequently present with atypical complaints, tend to wait longer before seeking medical attention [3] and have more extensive LV damage from myocardial infarctions [4, 7–9]. Nevertheless, published data on cardiac recovery and remodeling in older patients remain limited. Ennezat et al. reported similar rates of LV remodeling across age groups after acute anterior MI [8]. Importantly, this study excluded patients aged > 85 years and less than a third received primary percutaneous coronary intervention [8]. In contrast, Carrabba et al. demonstrated less improvement of LVEF and more adverse remodeling six months after STEMI in patients aged > 70 years who were treated by primary percutaneous coronary intervention [10]. We expand on these findings with data from a large, contemporary STEMI registry, showing an increased incidence of adverse LV remodeling, but a similar improvement of LV systolic function, in those ≥ 80 years of age.

Adverse LV remodeling in older patients may be explained by age-related changes in myocardial structure. First, the total number of cardiomyocytes decreases, along with hypertrophy in the remaining ones. Second, an increase in extracellular matrix volume and alterations in its composition, mediate fibrosis and subsequent remodeling [22, 23]. A study analyzing scar formation after MI in senescent mice observed a marked decrease in collagen deposition, with a reduced tensile strength of myocardial scar likely predisposing patients to increased LV dilation (adverse remodeling) [22]. These findings indicate that increased myocardial vulnerability with aging may manifest as greater susceptibility to adverse remodeling while preserving the ability to recover LV systolic function.

The larger increase in left atrial volume index we observed in older patients, might be ascribed to differing tissue characteristics, but not to worsening LV filling pressures, since greater improvement in LV filling pressures was noted in the older patient group. Importantly though, higher rates of new-onset atrial fibrillation during the first year after MI and increased use of anticoagulation were noted, suggesting that atrial fibrillation-related left atrial remodeling is the more likely mechanism of adverse left atrial remodeling. Given the high bleeding risk associated with anticoagulant use in older adults, our findings further support the need to implement strategies to reduce atrial remodeling.

### Anti-remodeling therapy in older patients

Both beta-blockers and renin-angiotensin-aldosterone system inhibitors have demonstrated effectiveness in ameliorating adverse, post-infarct remodeling [24, 25]. However, since older individuals have been excluded or underrepresented in most landmark trials [3], current guidelines recommend caution to extrapolate these conclusions to the elderly [3]. Physicians often hesitate to escalate treatment or discontinue current medications in older patients, not only due to the limited evidence base, but also because of concerns about polypharmacy and adverse effects [26]. While optimal medical therapy implementation after STEMI can be more complex due to transient LV dilation and dysfunction, simultaneous start of several blood pressure-lowering medications and intensive diuretic therapy resulting in negative fluid balance and hypotension [27]. In this setting, echocardiography, including quantitative Doppler data, might be useful in guiding therapy adjustment [27]. Older patients in our study presented with worse renal function and lower diastolic blood pressure. Both observations are relevant when deciding to start anti-remodeling therapy as they may indicate potential hemodynamic tolerance issues. Ennezat et al. showed similar use of beta-blockers and renin-angiotensin-aldosterone system inhibitors upon discharge across different age groups after MI, yet on follow-up older patients were prescribed beta-blockers less frequently [8]. In contrast, Perl et al. and Yudi et al. reported lower prescription rates of renin-angiotensin-aldosterone system inhibitors or angiotensin-converting-enzyme inhibitors, but no differences in beta-blocker use [16, 17]. We observed significantly lower prescription rates of beta-blockers and renin-angiotensin-aldosterone system inhibitors at discharge in older patients, with further decreasing use in both age groups (≥/< 80 years) at one-year follow-up. Nevertheless, in our population, prescription rates of these medications in older patients remained comparable to or higher than previously reported, with 80.7% and 86.7% receiving beta-blockers and renin-angiotensin-aldosterone system inhibitors at one year, respectively.

### Outcomes and clinical implications

Current guidelines advise against using advanced age as a reason to withhold primary percutaneous coronary intervention in STEMI [3]. Other than that, most questions about treatment strategies of older patients have not been tested in trials, and decision-making remains in the hands of treating physician [28]. All patients in our study received primary percutaneous coronary intervention, which might explain the relatively high survival rates [4, 5]. Nevertheless, older patients experienced six times higher five-year mortality rates, likely due to several contributing factors. First, increasing age is independently associated with all-cause mortality [3, 29]. Second, older patients tend to have a higher burden of cardiac and non-cardiac comorbidities (which was also observed in our cohort, and included pacemaker implantation and new-onset atrial fibrillation) [6]. Third, lower baseline LVEF post-infarct remains a well-established risk factor for adverse events [3]. Of note, while adverse LV remodeling is not independently associated with mortality, we have previously shown that it is associated with higher rates of heart failure hospitalization [30]. Consistent with these findings, our study showed that older patients suffered more heart failure hospitalizations and were prescribed more diuretics and nitrates over time.

The high rates of new-onset atrial fibrillation and pacemaker implantation suggest that more frequent electrocardiographic surveillance may be appropriate in older patients. Medication prescription rates at discharge and one year indicate a focus on symptomatic, rather than disease-modifying care. Heart failure hospitalization was higher in the older group, which might have been reduced by more aggressive anti-remodeling pharmacotherapy. A relatively high loss to follow-up echocardiography was observed in the older group (35% versus 7.4% younger patients), which cannot be entirely accounted for by mortality (10.6% at one year in the older patient group). Rather it likely reflects practical barriers to attending appointments such as needing transportation or assistance, and more frequent preference for palliative care over active treatment in this population. Targeted, randomized trials to guide treatment decisions in older patients are required and should incorporate outcomes such as quality of life and functional status.

### Study limitations

The main limitations of this study are related to its single-center, retrospective design and a relatively low number of older patients. Data on frailty and the exact cause of death were not systematically available and are therefore not reported. Granular details on the indication for permanent pacemaker implantation were not recorded in the database from which the analysis was performed. Since the study included patients presenting with STEMI from 2005 to 2021, the use of sodium-glucose cotransporter 2 inhibitors, angiotensin receptor-neprilysin inhibitors and proprotein convertase subtilisin/kexin type 9 inhibitors was not reported. More patients from the older group were lost to follow-up – subsequently, follow-up echocardiography findings likely represent the more active, older patient group and cannot be extrapolated to all older patients, including extremely frail ones.

## Conclusions

STEMI patients ≥ 80 years of age experienced more adverse LV remodeling and higher rates of cardiovascular events like heart failure hospitalization as compared to younger patients yet received less anti-remodeling medication and more symptom-focused treatment. Despite this apparent handicap, older patients (≥ 80 years) demonstrated comparable cardiac function recovery to younger patients and better survival than previously been reported.

## Supplementary Information

Below is the link to the electronic supplementary material.

Supplementary Material 1

## Data Availability

The data that support the findings of this study are available from the corresponding author upon reasonable request.

## Abbreviations

LV: left ventricle
LVEF: left ventricular ejection fraction
LVGLS: left ventricular global longitudinal strain
MI: myocardial infarction
STEMI: ST-segment elevation myocardial infarction
