# Supplementary material for: ST-segment elevation myocardial infarction in older adults: echocardiographic characterization and outcomes in contemporary practice: STEMI in older patients
Source: Int J Cardiovasc Imaging. 2026 Jul 11;42(11):2097–106. doi: 10.1007/s10554-026-03775-w (PMC13745401; doi:10.1007/s10554-026-03775-w)
Supplement: Supplementary file 1 [file 10554_2026_3775_MOESM1_ESM.docx]

**SUPPLEMENT**

**Table S1.** **Baseline characteristics for older patients lost to follow-up**

| **Parameter** | **Lost to follow-up (46)** | **Present (86)** | **P value** |
| --- | --- | --- | --- |
| Age, years | 83.9 ± 3.2 | 82.7 ± 2.7 | 0.03 |
| Male, % | 26 (56.5%) | 52 (60.5%) | 0.66 |
| Arterial hypertension | 27 (61.4%) | 50 (58.1%) | 0.72 |
| Previous MI | 6 (13.0%) | 6 (7%) | 0.25 |
| History of CHF | 2 (4.3%) | 2 (2.3%) | 0.52 |
| Previous AF | 7 (15.2%) | 2 (2.3%) | 0.005 |
| Diabetes mellitus | 7 (15.2%) | 15 (17.4%) | 0.74 |
| Current smoker | 5 (10.9%) | 9 (10.5%) | 094 |
| COPD | 3 (6.5%) | 8 (9.3%) | 0.58 |
| LVEF, % | 45.1 ± 11.3 | 48.3 ± 8.5 | 0.07 |
| LVGLS, % | 11.99 ± 4.3 | 13.3 ± 3.9 | 0.09 |
| LAVI | 37.8 ± 16.7 | 29.9 ± 11.3 | 0.002 |
| RVFAC | 41.6 ± 8.7 | 42.9 ± 8.0 | 0.4 |
| TAPSE | 18.8 ± 4.2 | 19.4 ± 2.9 | 0.34 |

AF: atrial fibrillation; CHF: chronic heart failure; COPD: chronic obstructive pulmonary disease; LAVI: left atrial volume index; LVEF: left ventricular ejection fraction; LVGLS: left ventricular global longitudinal strain; MI: myocardial infarction; RVFAC; right ventricular fractional area change; TAPSE: tricuspid annular plane systolic excursion
